# Adaptation to pH and Role of PacC in the Rice Blast Fungus *Magnaporthe oryzae*

**DOI:** 10.1371/journal.pone.0069236

**Published:** 2013-07-16

**Authors:** Patricia Landraud, Sarah Chuzeville, Geneviève Billon-Grande, Nathalie Poussereau, Christophe Bruel

**Affiliations:** UMR 5240 - Microbiologie, Adaptation et Pathogénie; Université Lyon 1, CNRS, Bayer CropScience, Villeurbanne, France; University of Minnesota, United States of America

## Abstract

Fungi are known to adapt to pH partly via specific activation of the Pal signaling pathway and subsequent gene regulation through the transcription factor PacC. The role of PacC in pathogenic fungi has been explored in few species, and each time its partaking in virulence has been found. We studied the impact of pH and the role of PacC in the biology of the rice pathogen *Magnaporthe oryzae*. Conidia formation and germination were affected by pH whereas fungal growth and appressorium formation were not. Growth *in vitro* and *in planta* was characterized by alkalinization and ammonia accumulation in the surrounding medium. Expression of the *MoPACC* gene increased when the fungus was placed under alkaline conditions. Except for *MoPALF*, expression of the *MoPAL* genes encoding the pH-signaling components was not influenced by pH. Deletion of *PACC* caused a progressive loss in growth rate from pH 5 to pH 8, a loss in conidia production at pH 8 *in vitro*, a loss in regulation of the *MoPALF* gene, a decreased production of secreted lytic enzymes and a partial loss in virulence towards barley and rice. PacC therefore plays a significant role in *M. oryzae*’s biology, and pH is revealed as one component at work during interaction between the fungus and its host plants.

## Introduction

The filamentous fungus *Magnaporthe oryzae* is an ascomycete of large economical importance due to its devastating impact on rice, barley, millet and, increasingly, wheat in different parts of the world [1]. The blast disease caused by this fungus affects the aerial part of the host plants and results in decreased yields to complete harvest loss depending on the organ infected and the time of infection [2], [3].

Infection begins by the germination of a conidium on the plant surface and the rapid differentiation of the germ tube into a specialized cell named appressorium. Turgor generation in this dome-shaped and highly melanized cell mechanically drives the penetration of the plant via a narrow penetration peg. Following development of branched bulbous hyphae in the first invaded cell, propagation in neighboring cells likely involves plamodesmata and proceeds again via the production of a narrow peg and then that of bulbous hyphae, but high pressure is not required [4]. Invasive hyphae are sealed in a plant membrane and they sequentially grow into living plant cells, consistent with a biotrophic behavior [4]. A switch to necrotrophic development would finally lead to maceration of the plant tissues and the appearance of necrotic lesions from which emerge new conidia through asexual reproduction. When exactly this switch occurs, however, remains unclear.

Signal transduction and production of lytic enzymes or metabolites are considered as important actors in the biology of plant pathogenic fungi, including *M. oryzae* [5]–[14]. One particular signal transduction pathway present in fungi relates to the sensing and adaptation to pH. As compared to other microorganisms, fungi are capable of growth and development over wide pH ranges, and several species, including plant pathogens, can modulate the pH of their environment by secreting acids or alkali [15], [16]. Thus far, a single pH-signaling pathway has been identified whose composition seems conserved throughout the fungal kingdom. In *Aspergillus nidulans* where it has been most studied, this pathway involves seven proteins [17]. Two of these proteins, PalH and PalI, are putative membrane proteins, and PalH (seven predicted transmembrane helices) is proposed to carry the pH-sensing activity. Following PalH activation by alkaline pH, signaling would proceed via the coupling of the receptor to the arrestin-like PalF [18] and the subsequent connection to PalC in the cell sub-cortical region, in interaction with the ESCRT-III proteins Vps32 and Vps23 [19]. Also interacting with Vps32 are PalA and PalB. PalA binds to the transcription factor PacC while PalB belongs to the cysteine protease family. PacC is activated through a two-step proteolysis reaction that sequentially involves PalB and the proteasome [20], [21]. Lastly, truncated PacC translocates to the nucleus where activation of “alkaline” genes and repression of “acidic” genes can proceed [22].

The role of the pH-signaling pathway in the virulence of various pathogenic fungi has been evaluated through the study of *PACC* deletion strains [23]–[29]. Interestingly, such deletion has mostly negative effects on the fungus capacity at secreting metabolites or lytic enzymes, undergoing developmental switches, penetrating the host or invading the host tissues. The pH-signaling pathway hence seems to control several cell functions whose contribution is relevant to various infection processes. At last, some degree of specificity in the genes targeted by this pathway would allow adaptation to the different environmental niches that various hosts or host tissues represent [30], [31].

To our knowledge, the role of pH in the biology of *M. oryzae* has never been investigated, and whether sensing and signaling of this environmental factor plays a role during interaction of this fungus with its host plants is unknown. This study is a first contribution to answering these questions.

## Results

### Influence of pH on the Biology of M. Oryzae

**Figure 1:**
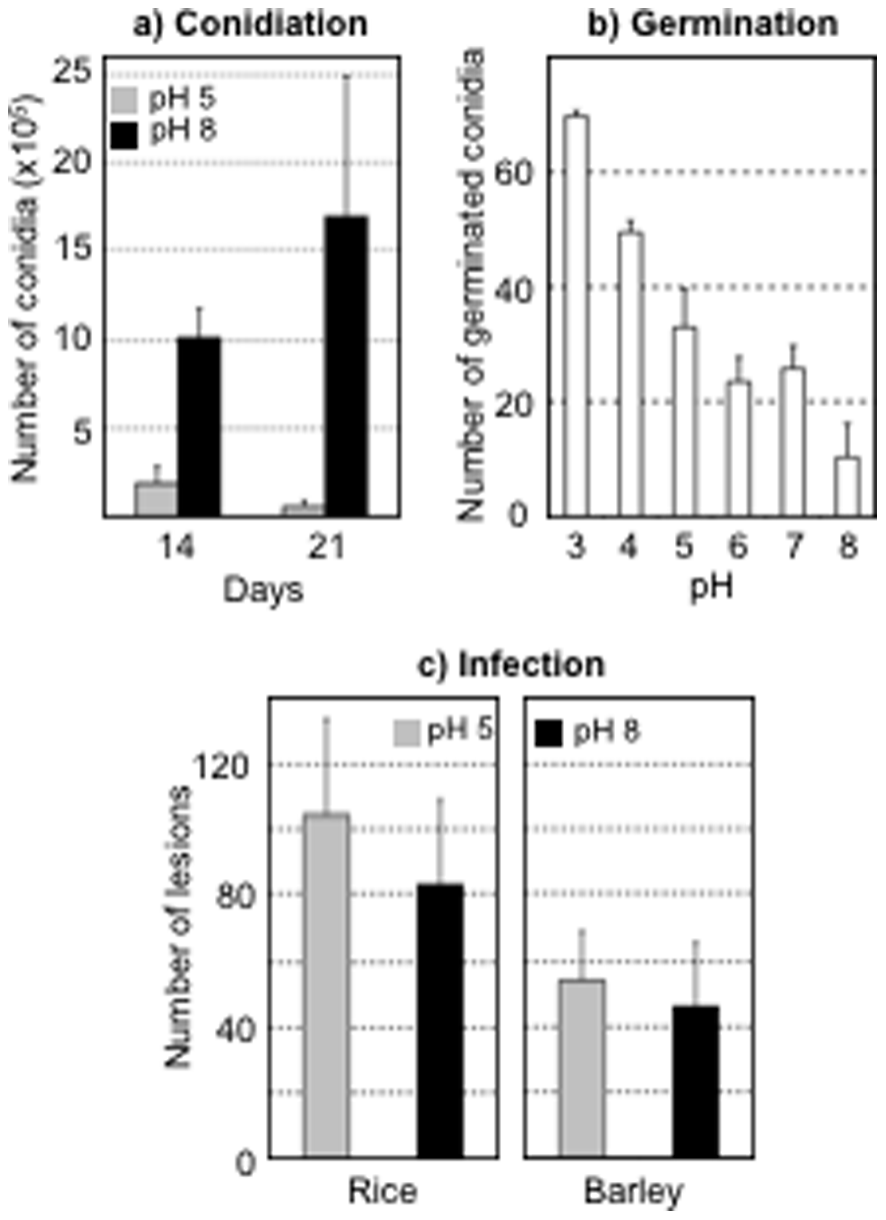
Influence of pH on the biology of *M. oryzae*. (a) The wild type strain Guy11 was grown for 14 and 21 days on solid rice medium buffered to pH 5 (grey) or to pH 8 (black) and conidia from one 9 mm plate were collected and counted under a microscope; Student test analysis of the data revealed a Pvalue of 0,004 at 14 days and of 0.07 at 21 days, due to higher variance. (b) One hundred conidia collected from cultures on non-buffered solid rice medium were incubated for 8 hours in water adjusted to pH 3 to 8, and their germination was monitored by microscopic observation of the presence of a germ tube at least 3 µm in length. (c) Conidia collected from cultures on solid rice medium buffered to pH 5 (grey) or pH 8 (black) were sprayed onto rice or barley plants, and the lesions on 10 separate leaves were counted after 5 days of incubation; Student test analysis of the data revealed a Pvalue of 0.078 for the barley experiment and of 0.079 for the rice experiment. All experiments were run in triplicates and standard deviations are shown.

The wild type Guy11 strain was grown on solid malt medium and then transferred to the same medium buffered to pH 5, 6, 7 or 8. The radial growth rates were measured and very similar results were obtained from pH 5 (0.6±0.1 cm/day) to pH 8 (0.63±0.1 cm/day). These results were unchanged when yeast extract or potato extract replaced malt in the culture medium (data not shown). Following such growth at pH 5 or at pH 8 for 14 days, counting of the collected conidia revealed a 5-fold higher production at pH 8 than at pH 5 (Fig. 1a). This was not due to a delay in conidiation at pH 5 since a similar pattern could be observed at 21 days. Germination of these conidia, conversely, decreased with pH, and this effect could be observed from pH 8 to pH 3 (Fig. 1b). Placed onto teflon membranes in drops of liquid medium adjusted to pH 5 or pH 8, these conidia did not differ in their capacity at forming a short hypha, and an appressorium after 24 hours; only longer hyphae connecting the conidia to the appressoria could be noticed at pH 8 (data not shown). Finally, conidia collected from colonies grown at pH 5 or at pH 8 caused similar numbers of lesions on rice or barley leaves (Fig. 1c). Altogether, these results show that some distinct steps of *M. oryzae*’s biological cycle are influenced by pH and prompted us to select pH 5 and pH 8 as acidic and alkaline conditions for the rest of the *in vitro* studies.

### M. Oryzae Modulates the pH of its Environment

**Figure 2:**
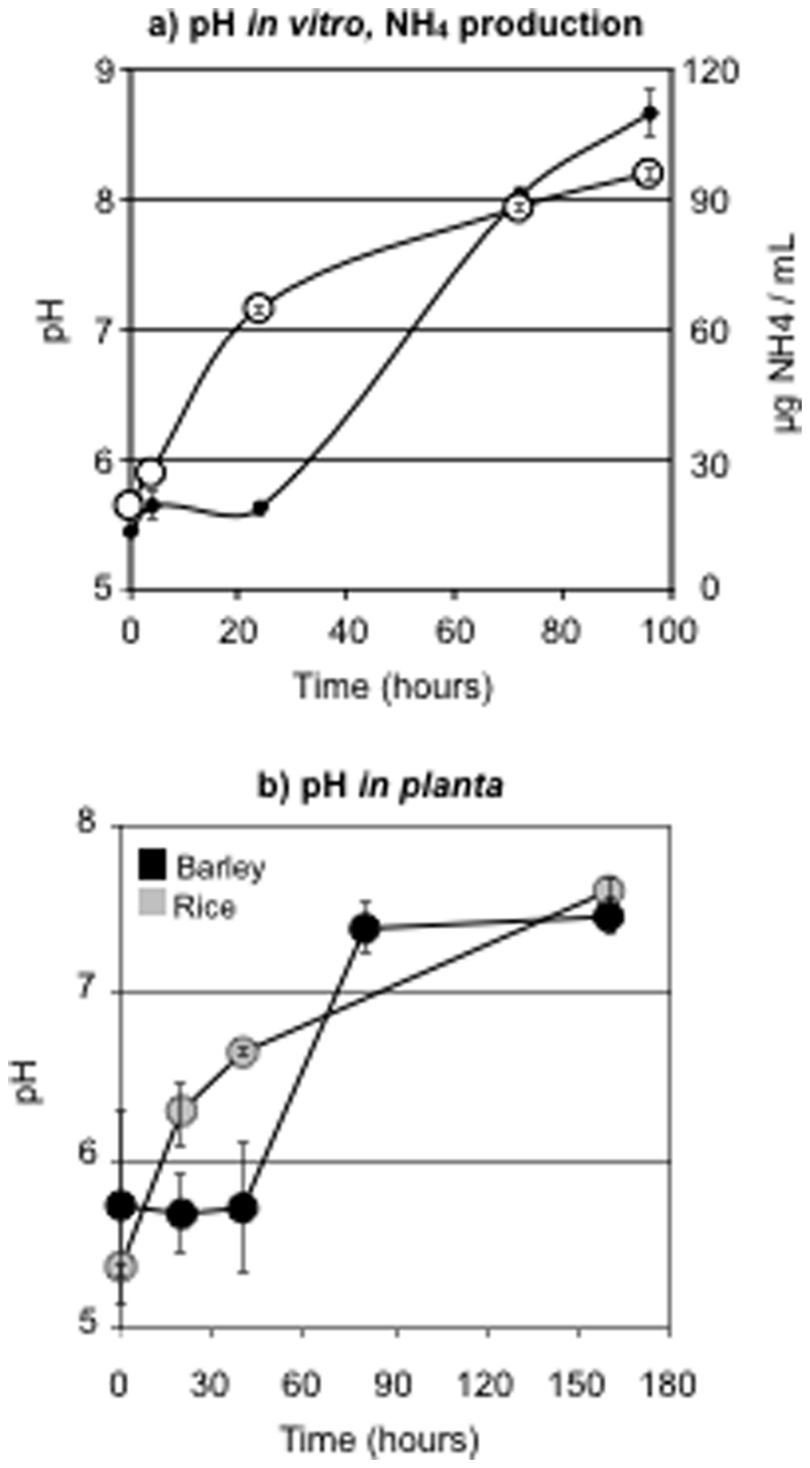
*M. oryzae* modulates its environmental pH. (a) pH of the medium (white circles) and ammonia accumulation (small black circles) during growth of the wild type strain Guy11 in liquid non-buffered TNK-YE medium. (b) pH of the infection zone during plant infection by the wild type Guy11 strain; detached barley (black) or rice (grey) leaves were infected with drops of conidia and the pH was recorded over time using a surface electrode. Results are the average of three independent experiments and standard deviations are shown.

The wild type Guy11 strain was grown in different liquid media and pH was monitored over time. When a complex medium containing yeast extract (TNK-YE) was used, a rise of about three pH units was observed in 96 hours (from pH 5.6 to pH 8.2) and a parallel accumulation of ammonia could be measured (Fig. 2a). When rice or barley extracts were provided as nutrients, alkalinization was also observed as the pH of the medium increased from 6.5 to 7.5 in 72 hours (data not shown). Lastly, an increase in pH was also observed during conidia-derived infection of both barley and rice detached leaves; the pH rose by about two units and reached pH 7.5 between 72 and 144 hours post-inoculation (Fig. 2b).

### Conservation of the pH-signaling Pathway Genes in M. Oryzae and Gene Expression Analysis

**Figure 3:**
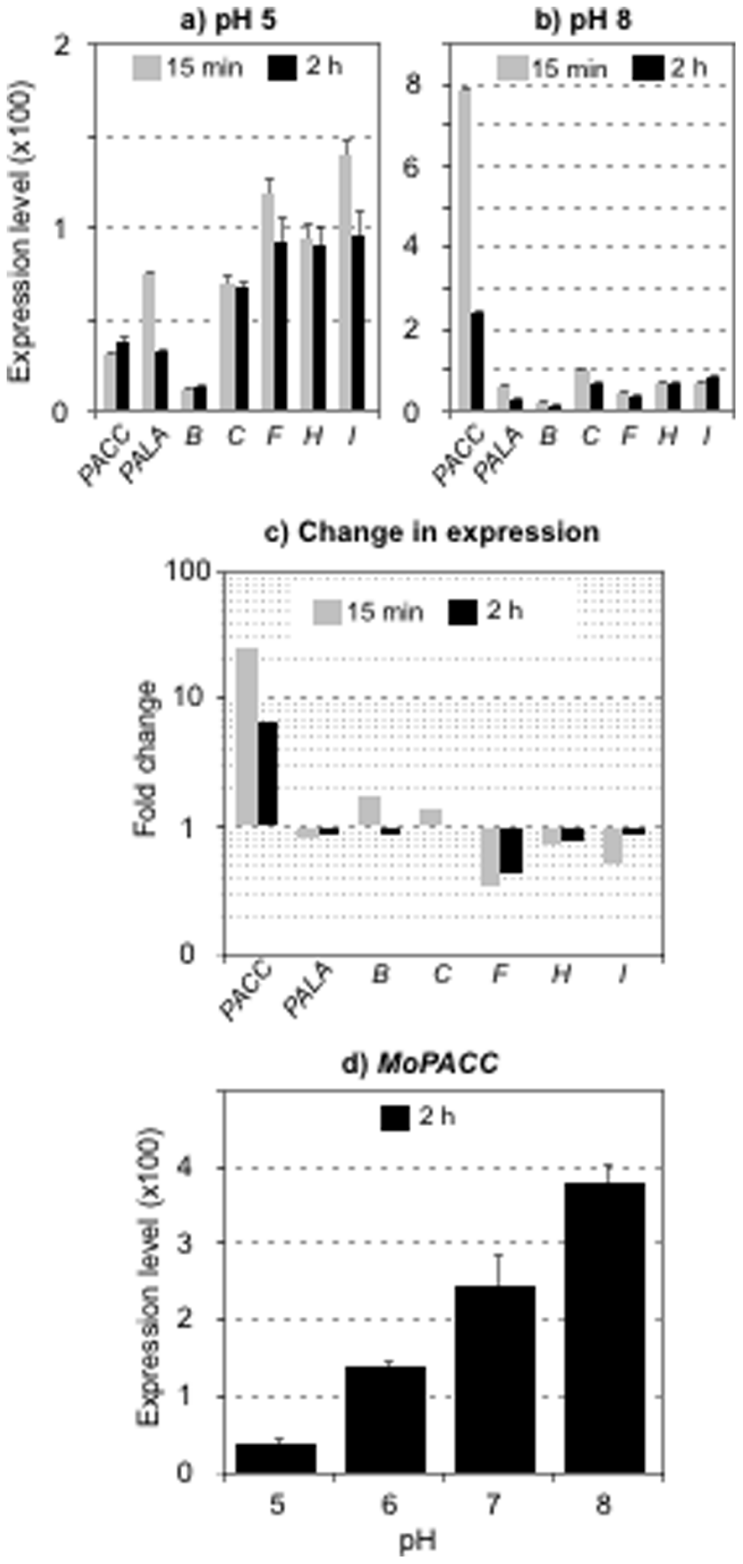
Expression of the *M. oryzae* pH-signaling related genes. Following 15 minutes (grey) or 2 hours (black) transfer of the wild type Guy11 strain from non-buffered liquid medium to fresh medium buffered to pH 5 or pH 8, total mRNA was extracted and quantitative PCR analysis was carried out using primers specific of the *MoPACC* and all the six *MoPAL* genes (Table S1). Quantification was based on the 2^ΔCT^ method using the *MoEF1*α gene as reference. Three independent biological replicates were analyzed for each studied gene. a) Gene expression after transfer to pH 5. b) Gene expression after transfer to pH 8. c) Change in expression between pH 5 and pH 8 (ratios are plotted using a logarithmic scale). d) *MoPACC* expression following 2 hours transfer of the fungus to media buffered to different pH values.

The *A. nidulans* PalA, PalB, PalC, PalF, PalH, PalI and PacC protein sequences were used to perform BlastP searches of the Broad Institute *M. oryzae* protein database (http://www.broad.mit.edu). All the homologs in *M. oryzae* were easily identified due to strong conservation of each of the protein sequence between the two fungi (Table 1). The *PACC* gene homolog, hereafter named *MoPACC*, clusters with other fungal *PACC* genes in coherence with the expected fungal phylogeny (Fig. S1). The genome-based predicted *MoPACC* open reading frame was confirmed by reverse transcription of *MoPACC* mRNA, amplification and cloning of the cDNA and sequence analysis (data not shown). Expression of *MoPACC* and all the *MoPAL* genes was then measured. *M. oryzae* was cultured in a non-buffered medium (pH 5.5) and transferred 15 minutes and 2 hours to fresh media buffered to pH 5 or to pH 8. Following transfer to pH 5, quantitative PCR analysis showed a very low expression level for *MoPACC* and *MoPALB*, higher expression levels for *MoPalF*, *H* and *I*, and intermediate expression levels for *MoPALA* and *MoPALC* (Fig. 3a). Upon transfer to pH 8, the expression of *MoPACC* strongly increased (24-fold) in 15 minutes, and remained high (6-fold) two hours after transfer (Fig. 3b and 3c). An other significant change in expression was recorded for *MoPALF* after transfer to pH8; its expression dropped more than 2-fold both at 15 minutes and 2 hours post-transfer (Fig. 3b and 3c). The expression of all the other *MoPAL* genes did not change significantly at pH 8 (Fig. 3b and 3c). Finally, we observed a linear increase in *MoPACC* expression when the fungus was exposed to increasing pH values (Fig. 3d).

**Table 1:** *MoPACC* and *MoPAL* genes.

Gene name	Locus	Gene length (nt)	ORF length (nt)	Introns	Protein length (AA)	Identity (%)
*MoPALA*	MGG_00833.6	2776	2550	**3**; 9–81, 329–403,2368–2445	849	49
***MoPALB***	MGG_06335.6	2768	2694	**1**; 363–436	897	37
*MoPALC*	MGG_09311.6	1612	1524	**1**; 1252–1339	507	40.4
*MoPALF*	MGG_01615.6	2573	2475	**1**; 466–563	824	35.7
*MoPALH*	MGG_06440.6	2490	2490	none	829	34.1
*MoPALI*	MGG_02630.6	2355	2082	**3**; 200–268, 1689–1763,1968–2096	693	31.8
*MoPACC*	MGG_10150.6	2055	1680	**3**; 211–424, 629–705,797–880	559	34.9

The *M. oryzae PAL* and *PACC* genes are presented. Introns were searched using the Softberry software and both their number (bold) and positions (start-end) are given. The length of the corresponding predicted proteins and the identity of these proteins to their *A. nidulans* counterparts is provided.

### Deletion of the M. oryzae PACC Gene

**Figure 4:**
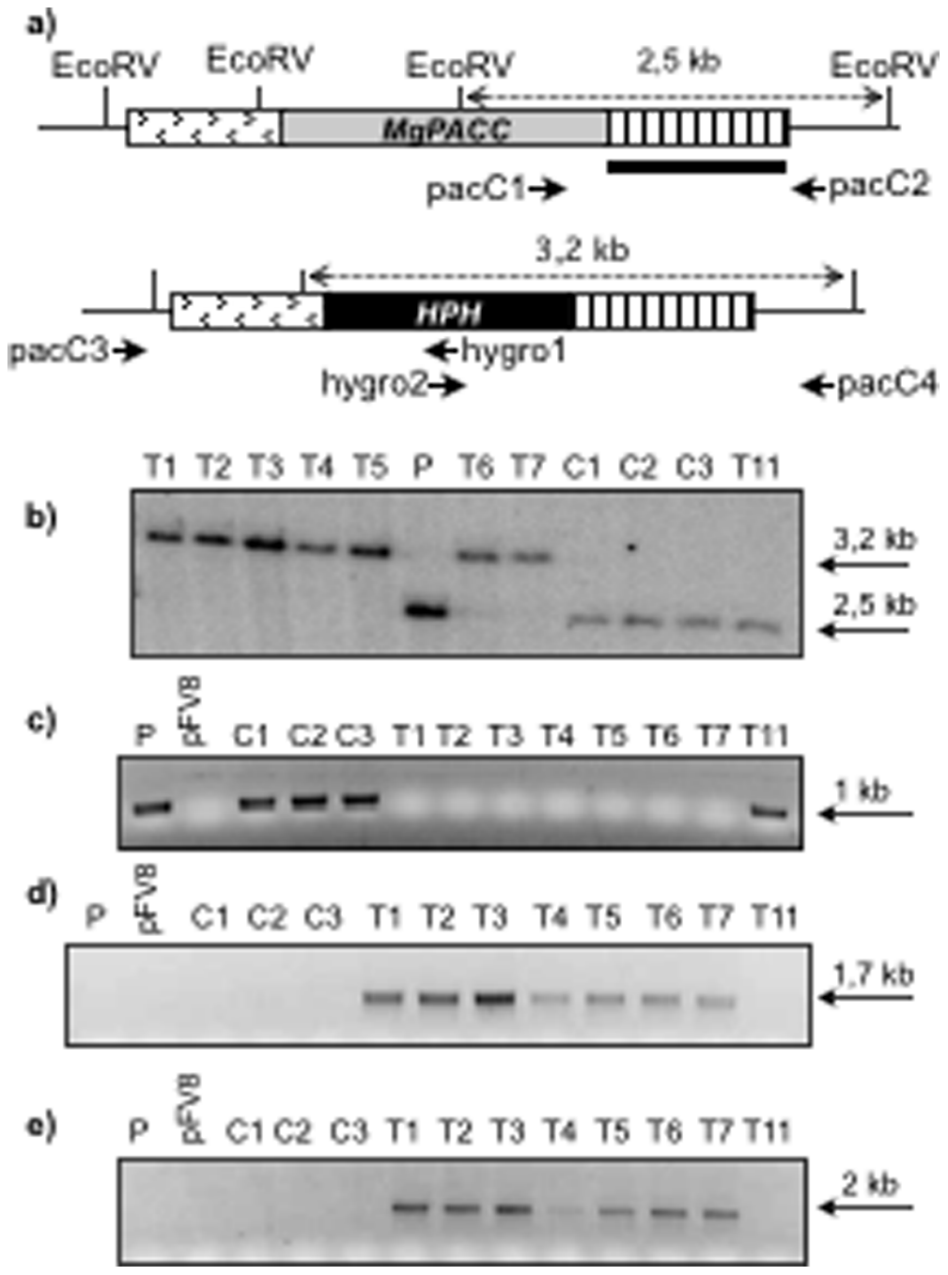
Deletion of the *MoPACC* gene. (a) Schematic representation of the *MoPACC* gene replacement by the hygromycin resistance gene flanked by 0.9 kb of downstream and 1.1 Kb of upstream sequences from the *MoPACC* locus. Primers (short arrows; Table S1) used for PCR analysis of the transformants are indicated together with the probe (black bar) used for Southern analysis and distances between the EcoRV restriction sites (dotted double arrow). (b) Southern analysis of the parental (P) strain, three control transformants (C1–C3; transformed with plasmid pFV8 carrying a *HPH*-only DNA cassette) and eight transformants of interest (T1–T11) using the DNA probe shown in (a). (c) PCR amplification of *MoPACC* using primers pacC1 and pacC2. (d) PCR amplification using pacC3 and hygro1. (e) PCR amplification using pacC4 and hygro2.

To delete *MoPACC,* we constructed a gene replacement cassette in which the *HPH* resistance gene was flanked by 0.9 Kb of upstream and 1.1 Kb of downstream genomic sequences from the *MoPACC* locus (Fig. 4a). The cassette was introduced by protoplast transformation into the high-frequency-gene-replacement strain Guy11-Δku80 [32], and seventeen hygromycin-resistant transformants were collected; eight of these transformants were subjected to molecular analysis. Following purification by single-spore isolation, *MoPACC* replacement was examined by Southern blotting. As shown in figure 4b, the hybridization profiles were compatible with a single gene replacement event in seven strains (T1–T7). In transformant T11, the profile was identical to that of the parent strain. Using PCR, these results were confirmed by the lack of detection of the *MoPACC* gene in all T1–T7 strains, its detection in T11, the amplification of the integrated cassette’s left and right junctions in T1–T7, and the absence of these fragments in T11 (Fig. 4c-e). Finally, amplification of the *HPH* gene was positive in all transformants except T11 (data not shown). Transformants T1, T2 and T3 were selected for the rest of the study together with strain C2 (ectopic integration of a *HPH* gene-only DNA cassette). Similar results were collected for the T1, T2 and T3 strains, and those obtained in the T2 strain are presented below.

### Impact of MoPACC Deletion on Fungal Growth, Alkalinization and Expression of the MoPAL Genes

**Figure 5:**
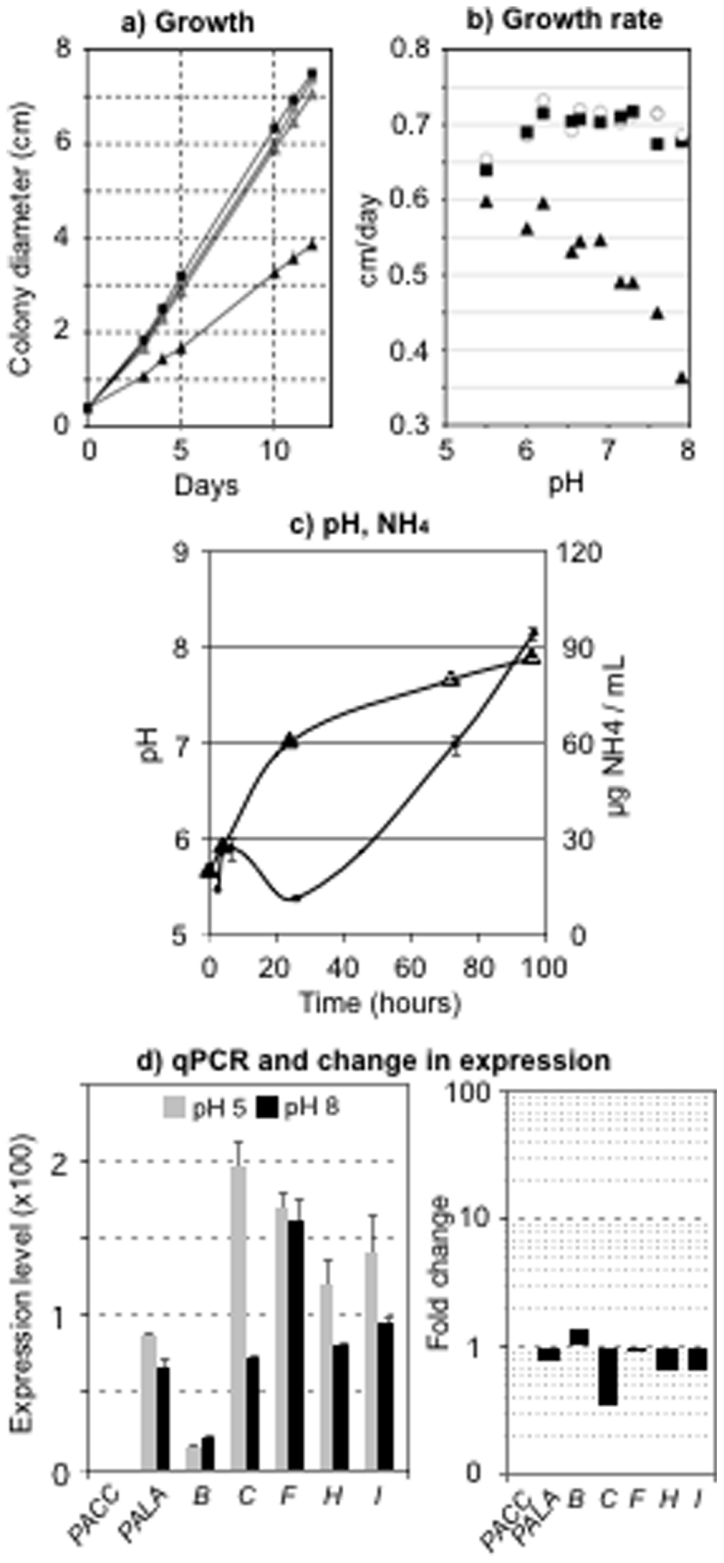
Impact of *MoPACC* deletion on the fungal growth and alkalinization. (a) Growth of the parental (square) and deletion (triangle) strains on solid medium buffered to pH 5 (grey) or pH 8 (black). (b) Growth rate as a function of pH in the parental (square), control (circle) and mutant (triangle) strains grown on 10 solid media buffered to 10 pH values ranging from 5.5 to 7.9. (c) pH of the culture (big triangles) and ammonia accumulation (small triangles) during growth of the deletion strain in liquid non-buffered TNK-YE medium. (d) Expression of the *MoPAL* genes in the *MoPACC* mutant strains. Following 15 minutes transfer of the deletion strain from non-buffered liquid medium to fresh medium buffered to pH 5 (grey) or pH 8 (black), total mRNA was extracted and quantitative PCR analysis was carried out using primers specific of the *MoPACC* and all the six *MoPAL* genes (Table S1). Three independent biological replicates were analyzed and quantification was based on the 2^ΔCT^ method using the *MoEF1*α gene as reference. The experiments were performed in parallel to those reported for the wild type strain in figure 3. Left panel : Gene expression after transfer to pH 5 or to pH 8. Right panel : Change in expression between pH 5 and pH 8 (ratios are plotted using a logarithmic scale).

The parental, control (C2) and deletion (T2) strains were inoculated onto solid medium either buffered to pH 5 or to pH 8 and their radial growth was monitored over time. Growths of the parental and control strains were identical at pH 5 and pH 8 (data not shown). Growth of the deletion strain was identical to that of the parental strain at pH 5, but dropped by 50% at pH 8 (Fig. 5a). We then used three buffer systems allowing pH overlaps to generate solid media ranging from pH 5.5 to pH 7.9, and we monitored growth rate. As shown in figure 5b, the growth rates of both the parental and control strains changed only slightly over the whole pH range tested. On the contrary, that of the deletion strain gradually and continuously decreased with increasing pH. When the deletion strain was grown in liquid culture, ammonia accumulation could be measured in the medium together with a rise in pH (Fig. 5c), but both the total amount of ammonia and the final pH value were slightly lower than those measured with the wild type strain (Fig. 2a). Finally, expression of *MoPACC* and all the *MoPAL* genes was measured in the deletion strain at pH 5 and at pH 8 (Fig. 5d). As expected, the expression of *MoPACC* could no longer be observed. Moreover, when compared to the wild type strain, the expression of *MoPALC* doubled at pH 5 and remained similar at pH 8 and the expression of *MoPALF* increased slightly at pH 5 and no longer dropped at pH 8.

### Impact of MoPACC Deletion on Asexual Reproduction and on Virulence

**Figure 6:**
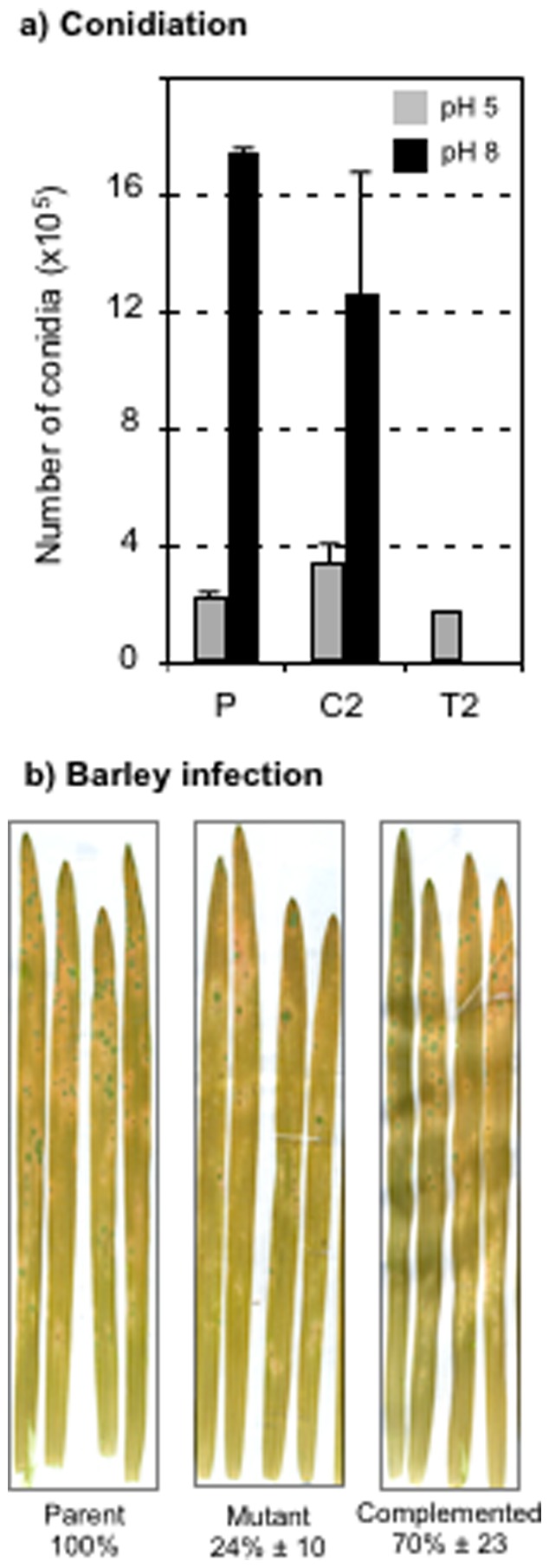
Impact of *MoPACC* deletion on the fungus biology. (a) Conidia production in the parental (P), control (C2 (ectopic)) and mutant (T2) strains grown for 14 days on solid rice medium buffered to pH 5 (grey) or to pH 8 (black). (b) Pathogenicity tests on barley leaves infected by conidia from the parent, mutant and complemented strains. Lesions on 10 separate leaves were counted after 5 days of incubation. All experiments were run in triplicates and standard deviations are shown.

A total defect in conidia production was monitored *in vitro* at pH 8 in the deletion strain when compared to the parental and control (ectopic) strains (Fig. 6a). At pH 5, conidiation still occurred, but a 30% decrease was nonetheless observed in comparison to the parental strain. None of these data resulted from a delay in conidiation in the deletion strain since identical results were obtained after longer culture time (7 more days; data not shown). The impact of *MoPACC* deletion on virulence was next analyzed. Barley leaves sprayed with conidia collected from the deletion strain exhibited about a fourth of the lesions counted on leaves infected by the parental or control strains (Fig. 6b). When the rice Sariceltic cultivar was used as host, lesions were also less numerous on the leaves infected by the mutant, but twice as much as that observed on barley (53.8±8%). Virulence was not delayed since identical results were observed after longer infection time (14 more days). The lesions looked identical on the leaves infected by the parent and mutant strains and, interestingly, conidia were similarly visible on the lesions caused by these two strains (data not shown). Lastly, when the rice Maratelli and CO-39 cultivars were used (more resistant towards *M. oryzae*), difference in virulence could no longer be observed between the parent and mutant strains.

### Complementation of the ΔpacC Mutant Strain

Complementation of the T2 deletion strain was performed via protoplasts transformation and the use of a DNA cassette carrying both the wild type *MoPACC* gene and the sulfonyl-urea resistance gene. One transformant was obtained and its capacity to grow like the parental strain at pH 8 and to produce conidia at pH 8 was restored to the level of the wild type strain (Table 2). Virulence towards barley leaves was restored to 70% of the wild type level (Fig. 6b). These results indicate that the phenotype of the studied *MoPACC* deletion strains most likely originates from the single genetic alteration of this gene.

**Table 2:** Results of the complementation studies.

Strain	Growth rate pH 8 (cm/day)	Conidia production pH 8 (conidia × 10^4^/plate)
Parent Δ*ku80*	0.67±0.02	94±27
	(100%)	(100%)
Mutant Δ*pacC* T2	0.28±0.03	1±1
	(42%)	(1%)
Δ*pacC*+*pacC*	0.64±0.01	103±18
	(95%)	(110%)

Growth and conidiation *in vitro* were measured for the complemented strain Δ*PACC+PACC* under the same conditions used for the study of the Δ*PACC* mutant strain (Fig. 5).

### Loss of Virulence and Host Specificity

**Figure 7:**
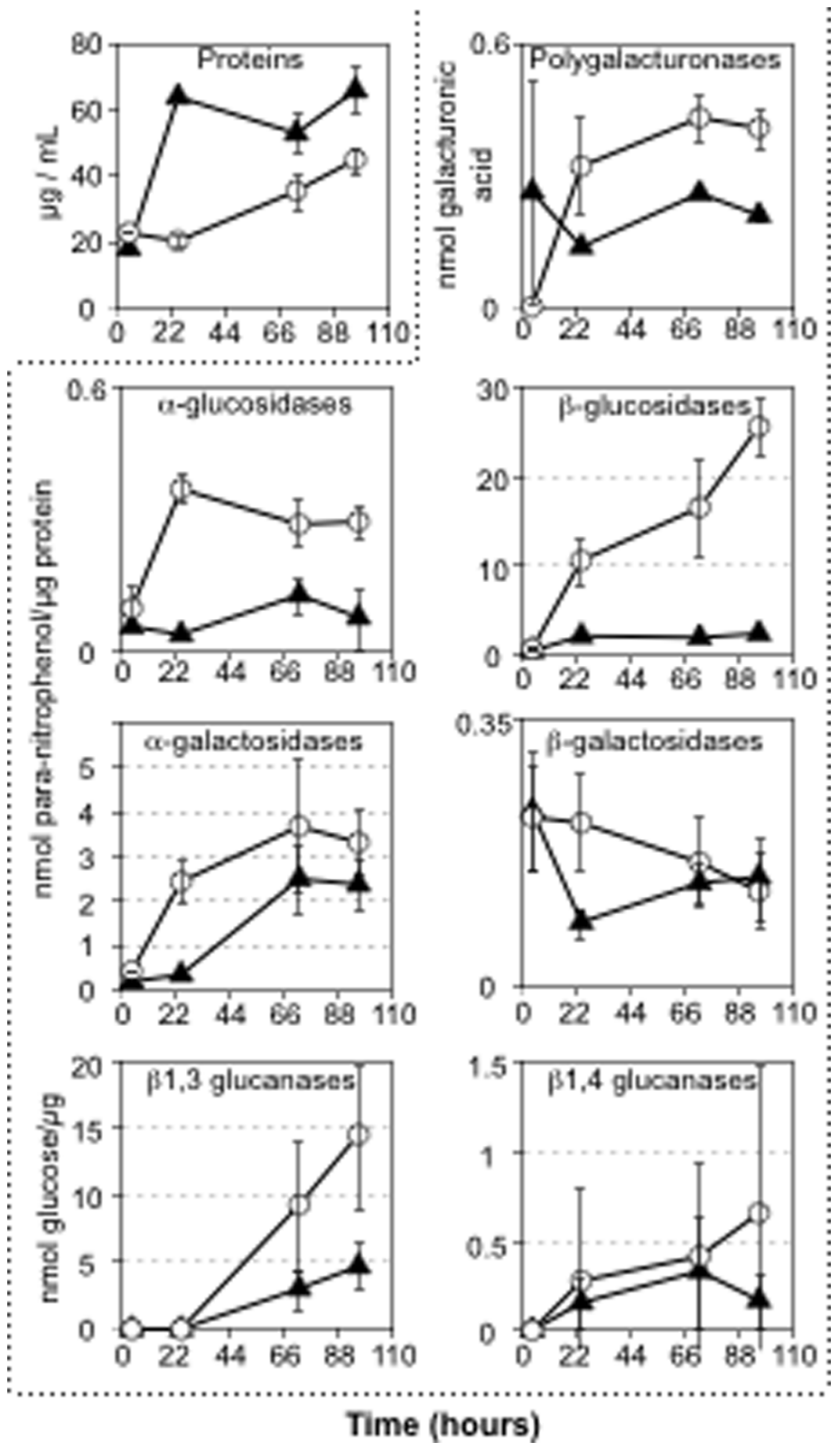
Impact of *MoPACC* deletion on the production of secreted lytic enzymes. The parental (circle) and mutant (triangle) strains were grown in liquid TNK-YE and the cultures were filtrated at 4, 24, 72 and 96 hours post-inoculation (X axis). Proteins present in the filtrates (top left panel) and seven different enzymatic activities were measured (inside dotted frame). For each assay, specific activities are shown as the average results of 4 experiments. Standard deviations are indicated.

In order to understand how infection of barley could be impaired in about 80% of the cases in the absence of *MoPACC*, three early steps of the infection process were analyzed using microscopy. First, appressorium formation on barley epidermis was compared in the parental and deletion strains, and both strains showed similar shape and number of appressoria produced under the conditions used. Penetration of the barley epidermis was then monitored, and no difference could be noted between the mutant and parental strains. Finally, colonization of the cells neighboring the penetration site was examined, and this also seemed unaffected by the deletion of *MoPACC*. In the absence of obvious defect during the early stages of infection, we questioned the capacity of the mutant at invading the plant tissues during its necrotrophic phase, and this was indirectly investigated by comparing the production of secreted lytic enzymes in the parent and mutant strains. The fungus was grown in liquid medium under non-buffered conditions and the culture media were collected over time to measure both the amount of proteins secreted and the activity of seven carbohydrates degrading enzymes. As shown in figure 7, the total amount of proteins found in the culture media were similar at 4 hours post-inoculation for both strains, but differed later with more proteins found in the mutant than in the parent culture medium. In contrast, five enzymatic activities out of seven were reduced in the mutant strain, namely polygalacturonases, α-glucosidases, β-glucosidases, α-galactosidases and β−1,3 glucanases.

## Discussion

This study explored the relationship between pH and the biology of *M. oryzae* through the particular scope of the fungal regulator PacC. First, a rise in pH during infection of either rice or barley, and a rise in pH together with ammonia production during fungal growth *in vitro* were monitored. Some plant pathogenic fungi like *S. sclerotiorum* and *B. cinerea* are known to acidify their environment during infection via the secretion of acids [15]. Others, like *Alternaria alternata* and several *Colletotrichum* species, tend to alkalinize the invaded plant tissues via the secretion of alkali like ammonia [33], [34]. This capacity at modifying the surrounding pH likely contributes to the advantageous adaptation of the pathogens to their host plants [30], and our results would support that *M. oryzae* be classified alongside *A. alternata* and *Colletotrichum*. Using confocal microscopy and the pH-sensitive fluorophore SNARF-1 [35], we attempted to measure micro-environmental pH changes in barley infected epidermis. We however failed at collecting solid data and we hence cannot provide information about putative alkalinization of individual cells at the early stage of infection.

Referring to the wild type Guy-11 strain that was used in this study, and under the nutrients and buffer conditions used, growth of *M. oryzae* occurs at the same rate over three pH units (5 to 8), and this illustrates a good adaptability to pH. In contrast, and interestingly, asexual reproduction and conidia germination are sensitive to modification of the surrounding pH. Indeed, pH 8 has a strong positive effect on the production of asexual spores whereas germination is favored at acidic pH values. With rice and barley surfaces being of mild acidic nature (see below), and with plant infection being characterized by alkalization (described above), this could represent a positive adaptation of the fungus infectious cycle to its natural environment. A similar proposition has recently been put forward for fungal dermatophytes [36].

The PacC-encoding gene sequence was found in *M. oryzae*’s genome, and it clustered with *N. crassa* and *C. globosum PACC* counterparts when performing phylogenic analysis of the available fungal *PACC* sequences (Fig. S1). Consistent with what has been shown in other fungi [29], [37]–[40], and consistent with the presence of three PacC consensus binding sites in its promoter sequence, *MoPACC* expression is activated when the fungus is placed under alkaline conditions. A 24-fold increase in expression was monitored after only 15 minutes of transfer from an acidic to an alkaline environment, and this demonstrates a very fast reaction of the fungal cell to pH that resembles what has been observed in *S. sclerotiorum* [40]. Homologs of the six *PAL* genes characterized in *A. nidulans* were also identified in *M. oryzae*. All but *MoPALF* exhibit low and pH independent expression like their *A. nidulans* counterparts [41]–[44]. The expression of *MoPALF* dropped when *M. oryzae* was placed under alkaline conditions, and this is reminiscent of results obtained in *S. cerevisiae* and *C. albicans* [45], [46], [39]. This regulation could not be observed in a *MoPACC* deletion strain and it is therefore likely mediated through PacC. With *PALF* encoding an arrestin-like protein necessary for alkaline pH signaling after putative activation of the membrane protein PalH [18], its down-regulation under these pH conditions could play a role in cell desensitization.

Through deletion of *MoPACC*, the role of the transcription factor PacC in the adaptation of *M. oryzae* to pH and during the infectious cycle could be assessed. The absence of *MoPACC* affects fungal growth at pH 8, and this can be reversed by complementation. It moreover affects optimal growth under both acidic and alkaline pH conditions, and our results show that pH adaptation fails gradually with increasing pH in the mutant strain. This result first indicates that the Pal/PacC signaling pathway operates in *M. oryzae* at both alkaline and acidic pH. Second, it could suggest that this signaling is less an on/off system than a continuum in which increasing amount of activated PacC would lead to increasing repression or activation of target genes as environmental pH increases. If PacC targets its own gene in *M. oryzae* like it does in other fungi, this hypothesis would be supported by a linear increase in *MoPACC* expression as a function of pH, and this was indeed observed. The continuum could originate from each cell within the multicellular hyphae increasing *MoPACC* expression with increasing pH or from increasing numbers of cells within the hyphae responding to pH with full transcription of *MoPACC.*

As reported for *PacC* mutant strains of *S. sclerotiorum* and *C. rosea* [24], [29], the virulence of *M. oryzae* was compromised by the absence of *MoPACC*, but it was not abolished. The deletion of *MoPACC* caused a reduction in the number of lesions occurring on leaves, but it did not modify the development of the lesions that formed. This suggests that PacC could be a dispensable, yet important, part of a threshold mechanism whose outcome would condition infection success or failure. Crossing of this threshold could be required during appressorium development, plant penetration, biotrophic growth or switch to necrotrophic growth. We did not observed that appressorium development or plant penetration was impaired in the mutant strain when tested on detached epidermis. In contrast, the production of several secreted enzymes was shown to be reduced in the mutant strain under *in vitro* growth conditions. If such production played a role in the invasion of the plant tissues during the necrotrophic phase, the hypothesized threshold mechanism could be part of the switching process between the biotrophic and necrotrophic stages. Complementation of the mutant strain fully restored fungal growth *in vitro*, but partly restored fungal virulence (70%). Such partial restoration could be due to partial restoration of lytic enzymes production, possibly caused by non optimal expression of the trans-gene in the complemented strain.

Interestingly, rice infection is less affected than that of barley by the absence of *MoPACC*. Since we showed that optimal adaptation to pH is increasingly dependent upon PacC as pH rises, this difference could be explained by barley representing a more alkaline environment than rice. This was indeed measured, both at the leaves surface (pH 5.35 for rice and pH 6.5 for barley) and in the leaves apoplasts (pH 5.2 for rice and pH 5.75 for barley). Besides pH, other unknown components could moreover be at work in barley, and not in rice, that could also solicitate *MoPACC* and make its presence more important for successful barley invasion than rice infection.

Finally, the absence of *MoPACC* seems to block conidia production at pH 8 *in vitro*, and this is fully restored by complementation. A reduced impact of the mutation (−30%) is seen under acidic conditions and conidia are produced on the plant lesions developed by the *MoPACC* mutant strain. Together with the effect of pH that we observed on conidiation in the wild type strain, this demonstrates that pH and its related signaling cascade play a role in asexual reproduction of *M. oryzae.* MoPacC could directly or indirectly target genes involved in conidiogenesis, and its bigger impact at pH 8 than at pH 5 is consistent with the higher expression of *MoPACC* under alkaline conditions. The production of conidia on plant tissues by the mutant strain however indicates that PacC is not a master regulator of conidiation *in planta*. Since the production of conidia is critical to the fungus propagation, and therefore to the spreading of rice blast epidemics, interference with MoPacC that would lead to reduced plant lesions and condiogenesis could be considered as a possible new approach to disease control.

In conclusion, pH is an environmental cue of significant importance in the biology of *M. oryzae,* in particular for conidia production and germination. During filamentous growth and conidiogenesis, MoPacC plays an important role under alkaline conditions but is also required under acidic conditions. During plant infection, MoPacC is essential for full virulence, possibly during transition from the biotrophic to the necrotrophic development phase.

## Materials and Methods

### Fungal Strains and Growth Conditions

The wild type *M. oryzae* isolate Guy11 pathogenic on rice and barley was obtained from the Centre de Coopération Internationale pour la Recherche Agronomique et le Développement (CIRAD). For sporulation, Guy11 was grown on 2% rice flour, 1% glucose, 0.2% KH_2_PO_4_, 0.3% KNO_3_, adjusted to pH 6 (rice medium). Other media used were: Malt-agar (Difco), potato-dextrose-agar (Oxoid) and TNK-YE derived from Tanaka-B medium [47] 1% glucose, 0.2% yeast extract, 0.2% NaNO_3_, 0.2% KH_2_PO_4_, 0.05% MgSO_4_·7H_2_O, 0.01% CaCl_2_·2H_2_O, 0.0004% FeSO_4_·7H_2_O, microelements as Tanaka-B, adjusted to pH 5.5–5.8. Media buffered to pH 5 contained 100 mM MES. Media buffered to pH 8 contained 100 mM MOPS. Hygromycin (Sigma-Aldrich, Saint-Louis, USA) was used at 0.012%. All solid media contained agar at 1.5%.

### Construction of the Gene Replacement Cassettes and Fungal Transformation

Upstream and downstream regions of the *MoPACC* gene were obtained by PCR using genomic DNA (100 ng) as template, primers pairs pacC3/pacC-left3’ and pacC4/pacC-right5’ and 0.5 units of DNA polymerase Phusion (Finnzymes, Espoo, Finland). The primers are listed in Table S1 and the strategy used for constructing the gene replacement cassettes is illustrated in Fig. 3a. Purified amplicons were digested by *Sfi*I whose restriction sites are present in the primers. The *HPH* cassette [48] conferring resistance to hygromycin was excised from plasmid pFV8 (pB2KS^-^ vector carrying the cassette flanked by *Sfi*Ia and *Sfi*Ib sites). Following ligation, the gene replacement cassette was obtained by amplification using primers pacC-left5’ and pacC-right3’. Fungal transformation was conducted as described in [32]. The pFV8 vector was used as control in transformation experiments. The DNA cassette designed for the complementation experiment was obtained by double-joint PCR [49] and directly used (3 µg of PCR product) to transform protoplasts. This cassette linked together the *MoPACC* sequence flanked by 0.9 Kb of its promoter and 0.2 Kb of its terminator and the sulfonyl urea resistance gene [50] also flanked by its promoter and terminator sequences.

### Molecular Analysis of the Transformants

Genomic DNA, prepared from lyophilised mycelium, as described in [51], and was used as template (100 ng) for PCR amplifications with primers described in Table S1; pacC1 and pacC2 were used to amplify *MoPACC* and hygro1, hygro2, pacC3 and pacC4 were used to amplify DNA fragments corresponding to the 5′ and 3′ cassette-locus junctions in transformants resulting from targeted gene replacement. For Southern hybridization, genomic DNA (5 µg) digested with *Eco*RV was subjected to 1% agarose gel electrophoresis, transferred to positive nylon membrane (Q-BIOgene, Irvine, USA) and hybridized at 65°C in Denhardt’s buffer with a ^32^P-labelled probe. The radioactive signals were revealed using a phosphoimager (Molecular Dynamics, Sunnyvale, USA).

### Quantitative PCR Analysis

Mycelium (100 mg) was frozen in liquid nitrogen, ground, and total RNA was extracted by phenol/chloroform separation and lithium chloride precipitation [52]. 20 µg of total RNA of each sample were treated with DNAse I (Ambion) to remove genomic DNA. Quality of total RNA was verified using Agilent 2100 Bioanalyzer, Agilent RNA 6000 Nano reagents and RNA Chips from Agilent. Total cDNA were produced by treating 2.5 µg of DNaseI-treated total RNA with Thermoscript RT (Invitrogen, Carlsbad, USA) as described by the manufacturer. Q-PCR experiments were performed on an ABI PRISM 7900HT (Applied Biosystems, Foster City, USA) using primers specific of the genes of interest (Table S1), using the Power SYBR® Green PCR Master Mix (Applied Biosystems), and according to the manufacturer’s instructions. Following examination of the primers efficiencies, the amplification reaction was carried out as follows: 95°C 10 min, 95°C 15 s and 60°C 1 min (50 cycles), 95°C 15 s, 60°C 15 s and 95°C 15 s. Quantification was based on the 2^ΔCT^ method using the *MoEF1*α or *MoILV5* gene as reference; the expression profiles of *MoPACC* and the *MoPAL* genes were identical when using either of these two genes.

### Phenotypic Analysis

*Radial growth* was measured daily following central inoculation of solid media with calibrated mycelium plugs (4 mm diameter) and incubation at 26°C in the dark. *Conidia production* was measured by counting conidia under a microscope after collecting them from 10–14 days old mycelium cultures grown on sporulation media. *Germination* was assayed by using 10^3^ spores/ml suspensions deposited onto 3% agar plates, and by counting conidia showing germ tubes under a stereomicroscope after 16h of incubation at 26°C. *Appressorium formation* was assayed by depositing 10^4^ spores/ml suspensions in 0.01% hexandecandiol onto Teflon membranes, and by counting the appressoria under a microscope after 24 hours of incubation at 26°C. *Plant penetration and colonization* were assayed by depositing conidia onto detached barley epidermis and through microscopic observations 24 and 48 hours later, respectively. *Host plant infections* were measured by using seedlings from barley and rice cultivars cultivated for 10 and 21 days, respectively (70% relative humidity; 25°C day, 20°C night). 2-leaf (barley) and 3-leaf (rice) stage plants were fed with 15 ml ammonium sulphate (1.2%), sprayed with a suspension (10 mL/pot) of 3 10^4^ spores/ml (barley) and 1.5 10^4^ spores/ml (rice) containing 0.3% gelatine, incubated overnight in a humid chamber (25°C, 99% humidity, dark) and transferred to a greenhouse for 4 days (25°C, 50% humidity, 12h light). Lesions were scored on 30 leaves at 5 days after inoculation. *Growth medium alcalinization* was measured on aliquots (200 µl) collected from 25 ml cultures (TNK-YE medium, 26°C, 110 RPM) by using a pH mini-electrode. The cultures were inoculated with mycelium previously grown under identical conditions for 3 days, filtered, and blended in 10 ml sterile water (1 ml was used as inoculum). *Ammonia production* was measured on culture filtrates (10 µl to 2 ml) incubated 5 minutes at room temperature with 100 µl of 0.1 M Na/K tartrate and 100 µl of Nessler’s reagent (Fluka, Germany). Following centrifugation (10°C, 10 min, 10000 *g*), the supernate absorbance was measured (420 nm). Freshly prepared 1 M (NH_4_)_2_SO_4_ served as standard [52]. Control reactions contained no filtrate or no tartrate.

### Enzyme Assays

40 mycelial plugs (*φ* = 0.5 cm) from fresh cultures on rice-medium plates were used to inoculate TNK-YE liquid cultures (100 mL). After 48 hours at 26°C under agitation (110 rpm), the fungus was collected by centrifugation (2500 rpm, 15°C, 8 min), rinsed twice in 25 mL sterile water and blended on ice (4×15sec) in 20 ml sterile water. 3 mL of blended mycelium were used to inoculate 4×25 mL of fresh TNK-YE liquid medium. Following 4, 24, 72 and 96 hours culture at 26°C under agitation (110 rpm), these 25 mL were filtrated and stored at −20°C before use as starting material to measure protein concentrations [53] and enzymatic activities. α- and β-glucosidase and galactosidase activities were determined using *p*-nitrophenyl-(α or β)D-(glucopyranoside or galactopyranoside) or o-nitrophenyl-β-D-galactopyranoside as substrates. The assays (3 ml total volume) contained 1 ml of appropriate substrate (1 mg. ml^−1^), 50 µl to 1 ml of the culture filtrates, 1 ml of 100 mM citrate/phosphate buffer, pH 5 (glucosidase assays) or pH 4 (galactosidases), and were incubated for 1 h at 37°C. Following addition of 1 ml of 1 M Na_2_CO_3_, released *p*-nitrophenol was measured by spectrophotometry (400 nm) [54]. β-1,3 and β-1,4-glucanase activities were respectively determined by using laminarin (0.4 mg/mL) and carboxymethylcellulose (1.2 mg/mL) as substrate [55]. Culture filtrates (10 to 200 µl) and substrates were incubated for 1 h at 37°C in 2 ml of 100 mM citrate/phosphate buffer, pH 5. Reactions were stopped by addition of 2 ml TBC (100 mM Na-tetraborate, 100 mM boric acid and 0.1% cyanoacetamide) and heating (10 min, 100°C). Following cooling to room temperature, free glucose was detected by spectrophotometry (270 nm). Polygalacturonase activities were determined by using polygalacturonic acid (0.8 mg/mL) as substrate [56]. Culture filtrates (10 to 100 µl) were incubated for 30 min at 48°C with the substrate in 600 µl of 30 mM acetate buffer pH 4. Reactions were stopped by addition of 1.2 ml TBC and heating (10 min at 100°C). Following cooling to room temperature, free galacturonic acid was detected by spectrophotometry (270 nm). Control reactions were stopped before incubation.

### In Planta pH Measurements

10-day old barley and 21-day old rice leaves were cut, kept in a humid chamber at 26°C and inoculated with drops of conidia (350 in 35 µl for barley and 100 in 10 µl for rice). pH was measured (5 repetitions) with a flat contact electrode (Bioblock Scientific, France) placed onto the lesions for 2 minutes periods. To measure the pH value of apoplastic fluids (rice and barley), 15 plantlets were placed at 22°C during 45 minutes in a water-saturated room immediately after cutting halfway their fourth leaf of. Extruded apoplast (about 300 µl) was collected using a pipette.

### Bioinformatics and Genes Studied

Blast and protein domains searches were performed at the Broad Institute (*M. oryzae* database: http://www.broad.mit.edu), EBI (Interproscan: http://www.ebi.ac.uk/interproscan) and NCBI (Blast - nr: http://www.ncbi.nlm.nih.gov/blast/). Alignments were performed with ClustalW and displayed using Genedoc. Phylogenetic analyses were performed using Mega 3.1. The resulting tree was drawn using MEGA version 3.1. Numbers at each node indicates percentage of 1000 bootstrap replicates. The fungal species used in the study were *Fusarium oxysporoum, F. verticillioides, F. graminearum, Trichoderma reesei, Neurospora crassa, Chaetonium globosum, Botrytis cinerea, Sclerotinia sclerotiorum, Phaeosphaeria nodorum, Aspergillus nidulans, A. fumigatus, A. niger, Yarrowia lipolytica, Candida albicans, Ashbya gossypii, Sacharomyces cerevisiae* and *Ustilago maydis.*

### Statistical Analysis

The numbers of observations were identical for each of the independent samples. Data were expressed as mean / standard deviation (SD). For statistical analysis, normality of the raw was first tested by using a Shapiro-Wilk test. Variances of the samples were then compared and found different. A Student bilateral t test was then applied to the data sets. The tests were performed using R software commands.

## Supporting Information

Figure S1 **PACC phylogeny.** *PACC* phylogenic tree rooted using the *U. maydis PACC* sequence and showing the fungal species used in the analysis. (1) Sordariomycetes (2) Leotiomycetes (3) Dothideomycetes (4) Eurotiomycetes (5) Hemiascomycetes and (6) Basidiomycetes.
(TIFF)

Table S1 **Primers used in the study.**
(DOC)

## References

[1] Talbot NJ (2003) On the trail of a cereal killer: Exploring the biology of *Magnaporthe grisea*. Annu. Rev. Microbiol. 57: 177–202. 10.1146/annurev.micro.57.030502.090957 14527276

[2] Ekwamu A (1991) Influence of head blast infection on seed germination and yield components of finger millet. Trop. Pest Manag. 37: 122–123.

[3] Scardaci SC, Webster RK, Greer CA, Hill JE, Williams JF, et al.. (1997) Rice blast: a new disease in California. Agronomy Fact Sheet Series 1997–2, Department of Agronomy and Range Science, University of California, Davis, USA.

[4] Kankanala P, Czymmek K, Valent B (2007) Roles for rice membrane dynamics and plasmodesmata during biotrophic invasion by the blast fungus. The Plant Cell. 19: 706–724. 10.1105/tpc.106.046300 PMC1867340 17322409

[5] Zhao X, Kim Y, Park G, Xu JR (2005) A mitogen-activated protein kinase cascade regulating infection-related morphogenesis in *Magnaporthe grisea*. Plant Cell. 17: 1317–1329. 10.1105/tpc.104.029116 PMC1088005 15749760

[6] Rho HS, Jeon J, Lee YH (2009) Phospholipase C-mediated calcium signalling is required for fungal development and pathogenicity in *Magnaporthe oryzae*. Mol Plant Pathol. 10: 337–346. 10.1111/j.1364-3703.2009.00536.x PMC6640429 19400837

[7] Dean RA, Talbot NJ, Ebbole DJ, Farman ML, Mitchell TK, et al (2005) The genome sequence of the rice blast fungus *Magnaporthe grisea* . Nature 434: 980–986. 15846337 10.1038/nature03449

[8] Li Y, Yan X, Wang H, Liang S, Ma WB, Fang MY, et al (2010) MoRic8 Is a novel component of G-protein signaling during plant infection by the rice blast fungus *Magnaporthe oryzae*. Mol Plant Microbe Interact. 23: 317–331. 10.1094/MPMI-23-3-0317 20121453

[9] Ramanujam R, Naqvi NI (2010) PdeH, a high-affinity cAMP phosphodiesterase, is a key regulator of asexual and pathogenic differentiation in *Magnaporthe oryzae*. PLoS Pathog. 6: e1000897. 10.1371/journal.ppat.1000897 PMC2865543 20463817

[10] Zhang H, Liu K, Zhang X, Song W, Zhao Q, et al (2010) A two-component histidine kinase, MoSLN1, is required for cell wall integrity and pathogenicity of the rice blast fungus *Magnaporthe oryzae*. Curr Genet. 56: 517–528. 10.1007/s00294-010-0319-x 20848286

[11] Liu W, Zhou X, Li G, Li L, Kong L, et al (2011) Multiple plant surface signals are sensed by different mechanisms in the rice blast fungus for appressorium formation. PLoS Pathog. 7: e1001261. 10.1371/journal.ppat.1001261 PMC3024261 21283781

[12] Takeda T, Takahashi M, Nakanishi-Masuno T, Nakano Y, Saitoh H, et al (2010) Characterization of endo-1,3–1,4-β-glucanases in GH family 12 from *Magnaporthe oryzae*. Appl Microbiol Biotechnol. 88: 1113–1123. 10.1007/s00253-010-2781-2 20680265

[13] Van Vu B, Itoh K, Nguyen QB, Tosa Y, Nakayashiki H (2012) Cellulases belonging to glycoside hydrolase families 6 and 7 contribute to the virulence of *Magnaporthe oryzae*. Mol. Plant Microbe Interact. 25: 1135–41. 10.1094/MPMI-02-12-0043-R 22852807

[14] Collemare J, Pianfetti M, Houlle A-E, Morin D, Camborde L, et al (2008) *Magnaporthe grisea* avirulence gene ACE1 belongs to an infection-specific gene cluster involved in secondary metabolism. New Phytol. 179: 196–208. 10.1111/j.1469-8137.2008.02459.x 18433432

[15] Verhoeff K, Leeman M, van Peer R, Posthuma L, Schot N, et al (1988) Changes in pH and the production of organic acids during colonization of tomato petioles by *Botrytis cinerea*. J. Phytol. 122: 327–333.

[16] St Leger RJ, Nelson JO, Screen SE (1999) The entomopathogenic fungus *Metarhizium anisopliae* alters ambient pH, allowing extracellular protease production and activity. Microbiology 145: 2691–2699. 10537191 10.1099/00221287-145-10-2691

[17] Peñalva M A, Tilburn J, Bignell E, Arst HN (2008) Ambient pH gene regulation in fungi: making connections. Trends Microbiol. 16: 291–71. 10.1016/j.tim.2008.03.006 18457952

[18] Herranz S, Rodriguez JM, Bussink HJ, Sanchez-Ferrero JC, Arst HN, et al (2005) Arrestin-related proteins mediate pH signaling in fungi. Proc. Natl. Acad. Sci. USA. 102: 12141–12146. 10.1073/pnas.0504776102 PMC1189327 16099830

[19] Galindo A, Calcagno-Pizarelli AM, Arst HN Jr, Peñalva MÁ (2012) An ordered pathway for the assembly of fungal ESCRT-containing ambient pH signalling complexes at the plasma membrane. J. Cell Sci. 125: 1784–95. 10.1242/jcs.098897 PMC3346829 22344261

[20] Penas MM, Hervas-Aguilar A, Munera-Huertas T, Reoyo E, Penalva MA, et al (2007) Further characterization of the signaling proteolysis step in the *Aspergillus nidulans* pH signal transduction pathway. Eukaryot Cell. 6: 960–970. 10.1128/EC.00047-07 PMC1951515 17416893

[21] Hervas-Aguilar A, Rodriguez JM, Tilburn J, Arst HN, Penalva MA (2007) Evidence for the direct involvement of the proteasome in the proteolytic processing of the *Aspergillus nidulans* zinc finger transcription factor PacC. J. Biol. Chem. 282: 34735–34747. 10.1074/jbc.M706723200 17911112

[22] Mingot JM, Espeso EA, Diez E, Penalva MA (2001) Ambient pH signaling regulates nuclear localization of the *Aspergillus nidulans* PacC transcription factor. Mol. Cell. Biol. 21: 1688–1699. 10.1128/MCB.21.5.1688-1699.2001 PMC86715 11238906

[23] Davis D, Edwards JE, Mitchell AP, Ibrahim AS (2000) *Candida al*bicans RIM101 pH response pathway is required for host-pathogen interactions. Infect. Immun. 68: 5953–5959. 10.1128/iai.68.10.5953-5959.2000 PMC101559 10992507

[24] Rollins JA (2003) The *Sclerotinia sclerotiorum* pac1 gene is required for sclerotial development and virulence. Mol. Plant Microbe Interact. 16: 785–795. 10.1094/MPMI.2003.16.9.785 12971602

[25] Kim YT, Prusky D, Rollins JA (2007) An activating mutation of the *Sclerotinia sclerotiorum* pac1 gene increases oxalic acid production at low pH but decreases virulence. Mol. Plant Pathol. 8: 611–622. 10.1111/j.1364-3703.2007.00423.x 20507525

[26] You BJ, Choquer M, Chung KR (2007) The *Colletotrichum acutatum* gene encoding a putative pH-responsive transcription regulator is a key virulence determinant during fungal pathogenesis on citrus. Mol. Plant Microbe Interact. 20: 1149–1160. 10.1094/MPMI-20-9-1149 17849717

[27] Miyara I, Shafran H, Kramer Haimovich H, Rollins J, Sherman A, et al (2008) Multi-factor regulation of pectate lyase secretion by *Colletotrichum gloeosporioides* pathogenic on avocado fruits. Mol. Plant Pathol. 9: 281–291. 10.1111/j.1364-3703.2007.00462.x PMC6640356 18705870

[28] Hua X, Yuan X, Di Pietro A, Wilhelmus KR (2010) The molecular pathogenicity of Fusarium keratitis: a fungal transcriptional regulator promotes hyphal penetration of the cornea. Cornea. 29: 1440–1444. 10.1097/ICO.0b013e3181d8383a PMC2991523 20856109

[29] Zou C-GG, Tu H-HH, Liu X-YY, Tao N, Zhang K-QQ (2010) PacC in the nematophagous fungus *Clonostachys rosea* controls virulence to nematodes. Environ Microbiol. 12: 1868–1877. 10.1111/j.1462-2920.2010.02191.x 20236165

[30] Prusky D, Yakoby N (2003) Pathogenic fungi: leading or led by ambiant pH? Mol. Plant Pathol. 4: 509–516. 10.1046/j.1364-3703.2003.00196.x 20569410

[31] Selvig K, Alspaugh JA (2011) pH Response Pathways in Fungi: Adapting to Host-derived and Environmental Signals. Mycobiology. 39: 249–256. 10.5941/MYCO.2011.39.4.249 PMC3385132 22783112

[32] Villalba F, Collemare J, Landraud P, Lambou K, Brozek V, et al (2008) Improved gene targeting in *Magnaporthe grisea* by inactivation of MgKU80 required for non-homologous end joining. Fungal Genet. Biol. 45: 68–75. 10.1016/j.fgb.2007.06.006 17716934

[33] Eshel D, Miyara I, Ailing T, Dinoor A, Prusky D (2002) pH regulates endoglucanase expression and virulence of *Alternaria alternata* in persimmon fruit. Mol. Plant Microb. Interact. 15: 774–779. 10.1094/MPMI.2002.15.8.774 12182334

[34] Prusky D, McEveoy JL, Leverentz B, Conway WS (2001) Local modulation of host pH by *Colletotrichum* species as a mechanism to increase virulence. Mol. Plant Microb. Interact. 14: 1105–1113. 10.1094/MPMI.2001.14.9.1105 11551075

[35] Diéguez-Uribeondo J, Förster H, Adaskaveg JE (2008) Visualization of localized pathogen-induced pH modulation in almond tissues infected by *Colletotrichum acutatum* using confocal scanning laser microscopy. Phytopathology. 98: 1171–1178. 10.1094/PHYTO-98-11-1171 18943405

[36] Martinez-Rossi NM, Persinoti GF, Peres NT, Rossi A (2012) Role of pH in the pathogenesis of dermatophytoses. Mycoses. 55: 381–387. 10.1111/j.1439-0507.2011.02162.x 22211778

[37] Tilburn J, Sarkar S, Widdick DA, Espeso EA, Orejas M, et al (1995) The *Aspergillus* PacC zinc finger transcription factor mediates regulation of both acid- and alkaline-expressed genes by ambient pH. Embo J. 14: 779–790. 10.1002/j.1460-2075.1995.tb07056.x PMC398143 7882981

[38] Lambert M, Blanchin-Roland S, Le Louedec F, Lepingle A, Gaillardin C (1997) Genetic analysis of regulatory mutants affecting synthesis of extracellular proteinases in the yeast *Yarrowia lipolytica*: identification of a RIM101/pacC homolog. Mol. Cell Biol. 17: 3966–3976. 10.1128/mcb.17.7.3966 PMC232249 9199331

[39] Ramon AM, Porta A, Fonzi WA (1999) Effect of environmental pH on morphological development of *Candida albicans* is mediated via the PacC-related transcription factor encoded by PRR2. J. Bacteriol. 181: 7524–7530. 10.1128/jb.181.24.7524-7530.1999 PMC94210 10601210

[40] Rollins JA, Dickman MB (2001) pH signaling in *Sclerotinia sclerotiorum*: identification of a pacC/RIM1 homolog. Appl. Environ. Microbiol. 67: 75–81. 10.1128/AEM.67.1.75-81.2001 PMC92519 11133430

[41] Denison SH, Orejas M, Arst HN (1995) Signaling of ambient pH in *Aspergillus* involves a cysteine protease. J. Biol. Chem. 270: 28519–28522. 10.1074/jbc.270.48.28519 7499363

[42] Negrete-Urtasun S, Denison SH, Arst HN Jr (1997) Characterization of the pH signal transduction pathway gene palA of *Aspergillus nidulans* and identification of possible homologs. J. Bacteriol. 179: 1832–1835. 10.1128/jb.179.5.1832-1835.1997 PMC178903 9045850

[43] Denison SH, Negrete-Urtasun S, Mingot JM, Tilburn J, Mayer WA, et al (1998) Putative membrane components of signal transduction pathways for ambient pH regulation in *Aspergillus* and meiosis in *Saccharomyces* are homologous. Mol. Microbiol. 30: 259–264. 10.1046/j.1365-2958.1998.01058.x 9791171

[44] Negrete-Urtasun S, Reiter W, Diez E, Denison SH, Tilburn J, et al (1999) Ambient pH signal transduction in *Aspergillus*: completion of gene characterization. Mol. Microbiol. 33: 994–1003. 10.1046/j.1365-2958.1999.01540.x 10476033

[45] Lamb TM, Mitchell AP (2003) The transcription factor Rim101p governs ion tolerance and cell differentiation by direct repression of the regulatory genes NRG1 and SMP1 in *Saccharomyces cerevisiae*. Mol. Cell. Biol. 23: 677–686. 10.1128/MCB.23.2.677-686.2003 PMC151549 12509465

[46] Porta A, Ramon AM, Fonzi WA (1999) PRR1, a homolog of *Aspergillus nidulans* palF, controls pH-dependent gene expression and filamentation in *Candida albicans*. J. Bacteriol. 181: 7516–7523. 10.1128/jb.181.24.7516-7523.1999 PMC94209 10601209

[47] Ou SH (1985). Rice Diseases, second ed. CAB Int., Mycological Institute, Wallingford, UK.

[48] Sweigard JA, Chumley F, Carroll AM, Farrall L, Valent B (1997) A series of vectors for fungal transformation. Fungal Genet. News. 44: 52–53.

[49] Yu JH, Hamari Z, Han KH, Seo JA, Reyes-Dominguez Y, et al (2004) Double-joint PCR: a PCR-based molecular tool for gene manipulations in filamentous fungi. Fungal Genet Biol. 41: 973–981. 10.1016/j.fgb.2004.08.001 15465386

[50] Chung KR, Shilts T, Li W, Timmer LW (2002) Engineering a genetic transformation system for *Colletotrichum acutatum*, the causal fungus of lime anthracnose and postbloom fruit drop of citrus. FEMS Microbiol. Lett. 213: 33–39. 10.1111/j.1574-6968.2002.tb11282.x 12127485

[51] Böhnert HU, Fudal I, Dioh W, Tharreau D, Notteghem JL, et al (2004) A putative polyketide synthase/peptide synthetase from *Magnaporthe grisea* signals pathogen attack to resistant rice. Plant Cell. 16: 2499–2513. 10.1105/tpc.104.022715 PMC520948 15319478

[52] Verwoerd B, Dekker M, Hoekema A (1989) A small-scale procedure for the rapid isolation of plant RNAs. Nucleic Acids Research. 17: 2362. 10.1093/nar/17.6.2362 PMC317610 2468132

[53] Bradford MM (1976) A rapid and sensitive method for the quantification of microgram quantities of protein utilising the principle of protein-dye binding. Anal. Biochem. 72: 248–264. 10.1016/0003-2697(76)90527-3 942051

[54] Werber G, Ahlke E, Nowak-Göttl U, Jürgens H, Verspohl EJ, et al. (1997) Asparaginase activities *in vitro* are highly sensitive to different buffer conditions. In: Büchner, T. (Ed.), Acute Leukemias VI : Prognostic factors and treatment strategies Springer, Berlin, Heildeberg, S512–S516.

[55] Hebraud M, Fèvre M (1988) Characterization of glycoside and polysaccharide hydrolases secreted by the rumen anaerobic fungi *Neocallimastix frontalis*, *Sphaeromonas communis* and *Piromonas communis*. J. Gen. Microbiol. 134: 1123–1129.

[56] Teunissen MJ, Smits AAM, Op den Camp HJM, Huis HJ, Vogels GD (1991) Fermentation of cellulose and production of cellulolytic and xylanolytic enzymes by anaerobic fungi from ruminant and non-ruminant herbivorus. Arch. Microbiol. 156: 290–296. 10.1007/BF00263000 1793336

