# Supplementary figures and images for: Adaptation to pH and Role of PacC in the Rice Blast Fungus Magnaporthe oryzae
Source: PLoS One. 2013 Jul 16;8(7):e69236. doi: 10.1371/journal.pone.0069236 (PMC3712939; doi:10.1371/journal.pone.0069236)

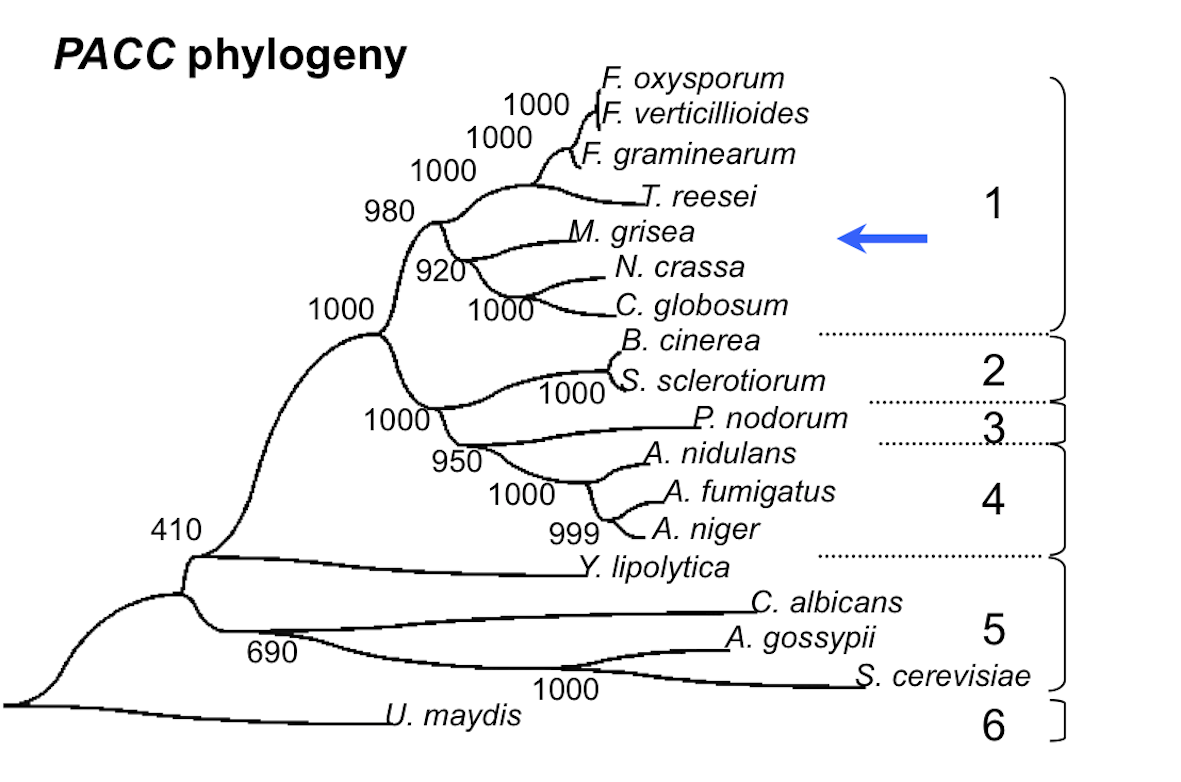

Supplement: Figure S1 — PACC phylogeny. PACC phylogenic tree rooted using the U. maydis PACC sequence and showing the fungal species used in the analysis. (1) Sordariomycetes (2) Leotiomycetes (3) Dothideomycetes (4) Eurotiomycetes (5) Hemiascomycetes and (6) Basidiomycetes. (TIFF) [file pone.0069236.s001.tiff]
