# Supplementary material for: Adaptation to pH and Role of PacC in the Rice Blast Fungus Magnaporthe oryzae
Source: PLoS One. 2013 Jul 16;8(7):e69236. doi: 10.1371/journal.pone.0069236 (PMC3712939; doi:10.1371/journal.pone.0069236)
Supplement: Table S1 — Primers used in the study. (DOC) [file pone.0069236.s002.doc]

Table S1: Primers used in the study

| Primer name | Sequence |
| --- | --- |
| pacC1 | CTGGCCCAGCACTACTACC |
| pacC2 | TATCTTAAGAGCACACGATACATTGCGC |
| pacCLB | TTTGGATATCAGTATGTCATACC |
| pacCleft3’ | CACGGCCTGAGTGGCCGCGAGCCACCCAGTCAAG |
| pacCRB | CACATCAACTATCTCAAGATCGC |
| pacCright5’ | GTGGGCCATCTAGGCCCGATGACGTCGAAGAGTCGG |
| pacCleft5’ | GTGAGCGTCGAGACCATCGG |
| pacCright3’ | AACACCGACTCCACGCTGCC |
| pacC3 | TATGAATTCTCATCAATCTGTATTGCGTCG |
| pacC4 | TATGAATTCAGCACACGATACATTGCGC |
| pacC5 | CAGGGAAACTCAGGGGTGG |
| pacC6 qPCR | GAGGGACCACATCACCTCTCA |
| pacC7 qPCR | TCACACTTGTGCGGCTTCA |
| hygro1 | AGGCTCTCGCTGAACTCCCCAATG |
| hygro2 | CATTGGGGAGTTCAGCGAGAGCCT |
| hygro3 | AGCCTGAACTCACCGCGACG |
| hygro4 | CGACCCTGCGCCCAAGCTGC |
| sulf1 | TGTATCGTGTGCTGAATTCATATATGACCATGATTACGCCAAGCG |
| sulf2 | CAACTGTTGGGAAGGGCGATC |
| sulf3 | GCAATTCCCGTGCAATAATCA |
| ef1α1 qPCR | GCCCGGTATGGTCGTTACCT |
| ef1α2 qPCR | AGCTGCTGGTGGTGCATCTC |
| palA1 qPCR | CCTTCCGGCAGCAAACG |
| palA2 qPCR | TGGTCCGCCAAACTGGAT |
| palB1 qPCR | GGCGCCGAACCCACATA |
| palB2 qPCR | TGGGCGTGGTGACTTTGAT |
| palC1 qPCR | GAAAATGCGTCCGATTCTGAA |
| palC2 qPCR | TGGAAACGCGCCAACAG |
| palF1 qPCR | GCCGAGCGGAGCAGAGA |
| palF2 qPCR | GGATAACGTGCTTGCAAATGG |
| palH1 qPCR | GCCGTGGCTCAGAAAGTACAG |
| palH2 qPCR | GGCAACGTTTGAGCCAAAGT |
| palI1 qPCR | TCCGAGGTTTGCTACTCCTGAT |
| palI2 qPCR | ACCAGCTTGTCCCGGAAC |
